# A Novel Glucose Isomerase from *Caldicellulosiruptor bescii* with Great Potentials in the Production of High-Fructose Corn Syrup

**DOI:** 10.1155/2020/1871934

**Published:** 2020-04-13

**Authors:** Chenxia Dai, Tingting Miao, Jinping Hai, Yunyi Xiao, Ying Li, Junren Zhao, Hulin Qiu, Bo Xu

**Affiliations:** ^1^Technology Research Center for Lingnan Characteristic Fruits & Vegetables Processing and Application Engineering of Guangdong Province, Food Science Innovation Team of Guangdong Higher Education Institutes, Guangdong University of Petrochemical Technology, Maoming, 525000 Guangdong, China; ^2^College of Biological and Food Engineering, Guangdong University of Petrochemical Technology, Maoming, 525000 Guangdong, China

## Abstract

Glucose isomerase (GI) that catalyzes the conversion of D-glucose to D-fructose is one of the most important industrial enzymes for the production of high-fructose corn syrup (HFCS). In this study, a novel GI (CbGI) was cloned from *Caldicellulosiruptor bescii* and expressed in *Escherichia coli*. The purified recombinant CbGI (rCbGI) showed neutral and thermophilic properties. It had optimal activities at pH 7.0 and 80°C and retained stability at 85°C. In comparison with other reported GIs, rCbGI exhibited higher substrate affinity (*K*m = 42.61 mM) and greater conversion efficiency (up to 57.3% with 3M D-glucose as the substrate). The high catalytic efficiency and affinity of this CbGI is much valuable for the cost-effective production of HFCS.

## 1. Introduction

Glucose isomerase (GI), also known as xylose isomerase, catalyzes the reversible isomerization of D-xylose and D-glucose to D-xylulose and D-fructose, respectively. In the food industry, GI is widely used in the industrial production of high-fructose corn syrup (HFCS) [1], which is a mixture of glucose and fructose and has the advantages of sweetness, low cost, and high solubility [2]. HFCS has become an increasingly common food ingredient in the last 40 years and is used extensively in various industrial fields, including the food, detergent, and pharmaceutics. According to the fructose content, HFCS is divided into three types: HFCS-42 (42% fructose, 53% glucose, and 5% polysaccharide), HFCS-55 (55% fructose, 42% glucose, and 3% polysaccharide), and HFCS-90 (90% fructose, 9% glucose, and 1% polysaccharide) [3]. The three types of HFCS have different uses in food production. HFCS-42 is the most popular type that is used in beverages, processed foods, cereals, and baked goods. HFCS-55 has the same level of sweetness as sucrose and a larger market demand than HFCS-42, which is used in soft drinks, ice cream, frozen desserts, and other sweetened beverages. HFCS-90 which is mainly used to make HFCS-55. [3]. Therefore, HFCS-55 is the most commonly used type; its commercial production requires complex processes including chromatography, purification, and concentration [4]. Therefore, it is of great importance to produce HFCS-55 in a cost-effective and one-step way [5].

It has been reported that a thermodynamic equilibrium exists between the isomerization reactions of glucose and fructose. Along with the increase of reaction temperature, the conversion rate of glucose to fructose also gradually increases. Thus, thermostable GI is more favorable for the one-step production of HFCS-55. If this thermostable GI can convert high concentrations of glucose to fructose at a higher rate, it would be remarkable for industrial purposes [1]. So far, a few high-temperature active GIs have been reported. For example, the GI from *Thermobifida fusca WSH03-11* produced in *Escherichia coli* had the maximum conversion rate of 45% (*w*/*v*) at pH 7.5 and 70°C [6], while the GI from *Thermus oshimai* (ToGI) has an optimum temperature of 95°C and at a maximum conversion rate of 52.16% at 85°C [7]. However, the conversion rates of these GIs are much lower than 55%, which is the limit of their industrial application.

The thermostable GI from the genus of *Caldicoprobacter* has been reported with good properties for HFCS-55 production [8]. In order to search for a novel glucose isomerase with higher conversion rate, we studied the putative GI gene from the thermophilic anaerobic *Caldicoprobacter bescii* DSM 6725. The gene was cloned and expressed in *E. coli BL21* (DE3). The enzyme showed the highest conversion rate of 57.3% with 3M D-glucose. Thus, it has great application potentials in the industrial production of HFCS-55.

## 2. Materials and Methods

### 2.1. Strains, Medium, and Chemicals

The strain *C. bescii* DSM 6725 was purchased from the Leibniz-Institut DSMZ-Deutsche Sammlung von Mikroorganismen und Zellkulturen GmbH (Braunschweig, Germany) and grown in DSMZ 516 medium. The Fast Pfu DNA polymerase and *E. coli* strains *BL21 (DE3)* and Trans1 were obtained from TransGen (Beijing, China). The *E. coli* cells were cultivated in *Luria-Bertani medium* (LB, 0.5% yeast extract, 1% tryptone, and 1% NaCl) supplemented with 100 *μ*g/mL kanamycin. The plasmids pEASY-T3 and pET-30a were used for cloning, sequencing, and expression, respectively. The substrate D-glucose from Sigma-Aldrich was used for the enzyme activity assay. Fructose and other chemicals were of analytical grade and commercially available.

### 2.2. Sequence Analysis and Gene Cloning

The *C. bescii* DSM 6725 genome sequence is now available (https://www.ncbi.nlm.nih.gov/genome/1747?genome_assembly_id=300706) and is known to encode for several functionally uncharacterized genes. An open reading frame (ORF), consisting of 1317 bp, coding for a putative xylose isomerase (GenBank accession number: ACM59729), was not characterized. Its nucleotide and amino acid sequences were analyzed by using the blastn, blastx, and blastp programs (https://blast.ncbi.nlm.nih.gov/Blast.cgi). The protein molecular weight and isoelectric point (pI) were performed using the Vector NTI Advance 11.5 software (Invitrogen). The genomic DNA of *C. bescii* DSM 6725 was extracted by using a TIANamp Genomic DNA Kit (Tiangen Biotech Co., China). Overlap PCR was used to construct the expression plasmid pET-CbGI. With the genomic DNA and expression vector pET-30a (+) as the templates, the CbGI-encoding gene and linear pET-30a were amplified by PCR with the specific primers containing overlapping fragments and C-terminal His6-encoding sequence presented in Table 1. The PCR products were cloned into the vector pEASY-T3 and transformed into *E. coli* Trans1 competent cells for sequencing. Transformants were selected on LB plates containing 100 *μ*g/mL ampicillin. The purpose gene *CbGI* ligated to pET-30a (+) expression vector with encoding C-terminal six-His sequence by overlapping methods.

### 2.3. Protein Expression and Purification

For CbGI production, the *E. coli BL21* cells harboring recombinant expression plasmid pET-CbGI were grown at 37°C in 300 mL LB medium containing 100 *μ*g/mL kanamycin to an OD600 of 0.5-0.6. The CbGI expression was then induced by 1 mM of isopropyl-*β*-D-1-thiogalactopyranoside (IPTG) at 30°C for 5 h. The *E. coli* cells were harvested by centrifugation (12,000 *g*, 10 min and 4°C) and suspended in 10 mL of buffer (500 mM NaCl, 20 mM phosphate buffer; pH 7.4). After cell disruption by sonication, the crude enzyme was collected by centrifugation (12,000 *g*, 10 min and 4°C) and mixed with histidine protein purification beads (Beaver, Suzhou, China) at 4°C for 2 h. A gradient of imidazole solution (40-300 mM) was applied to elute the recombinant protein. The eluates showing GI activities were pooled, dialyzed to remove the imidazole, and stored at 4°C. The protein concentration was determined by using the BCA protein assay kit (TransGen), with bovine serum albumin as the standard.

### 2.4. Enzyme Activity Assay

The GI activity was determined by measuring the amount of D-fructose. Unless otherwise stated, the standard reaction systems (1 mL) contained 0.5 mM of Co^2+^, 50 mM of D-glucose, and 0.05 mL of properly diluted purified CbGI in 50 mM of McIlvaine buffer (pH 7.0). The reactions were conducted at 80°C for 30 min and stopped by cooling in ice water. The amount of D-fructose produced was then determined by measuring the absorbance at 560 nm following the cysteine-carbazole-sulphuric acid method [8]. One unit of GI activity was defined as the amount of enzyme that produced 1 *μ*mol of D-fructose per min under the assay conditions.

### 2.5. Biochemical Characterization

The optimal pH for CbGI activity was determined in 50 Mm McIlvaine buffer (pH 3.0 to 8.0), Tris-HCl (pH 7.0 to 9.0), and glycine-NaOH (pH 9.0 to 12.0) at 80°C in for 30 min. The optimal temperature for CbGI activity was determined by performing the activity assays in Na_2_HP0_4_-NaH_2_PO_4_ buffer (pH 7.0) at temperatures ranging from 60°C to 95°C for 30 min. To estimate the pH stability, the enzyme was preincubated at 37°C for 1 h in buffers described above (pH 2.0–12.0), and the residual enzyme activities were then measured at standard conditions (80°C and pH 7.0 for 30 min). The thermal stability of CbGI was determined by measuring the residual enzyme activities under standard conditions after preincubation at various temperatures for 15 min, 30 min, and 60 min, respectively. All experiments were in triplicate.

### 2.6. Determination of Kinetic Parameters

To determine the kinetic parameters (*K*m, *V*max, *k*cat, and *k*cat/*K*m) of CbGI, the reaction systems (1 mL) performed using glucose at various concentrations from 10, 20, 40, 60, 80, and 100 mM at pH 7.0 and 80°C for 30 min. The reactions were terminated by cooling in ice water, and the amounts of D-fructose produced were measured by the HPLC chromatographic method using the column HPX-87C (250 × 4 mm) (BioRad, USA). The mobile phase, temperature and the flow rate were water, 50°C and 0.5 ml min^−1^, respectively. The experiment was carried out three times and each included triplicate. The kinetic parameters *K*m and *V*max were calculated from the Lineweaver-Burk plots.

### 2.7. Effect of Metal Ions and Chemical Reagents on CbGI Activity

The effects of metal ions and chemical reagents on the CbGI activity were determined by measuring the enzyme activities under standard conditions in the presence of various metal ions and chemicals (Co^2+^, Mn^2+^, Mg^2+^, Ni^2+^, K^+^, Cr^3+^, Na^+^, Zn^2+^, Fe^2+^, Ca^2+^, *β*-mercaptoethanol, SDS, and EDTA) at a final concentration of 10 mM.

### 2.8. Conversion Efficiency of CbGI

To determine the conversion rate of CbGI, reaction systems (3 mL) containing 1 M D-glucose, 50 mM phosphates buffer (pH 7.0), 0.5 mM Co^2+^, and 30 U, 60 U, or 90 U of CbGI were incubated at 80°C for different durations. Samples were taken periodically for the measurements of D-fructose contents. The conversion rate was calculated as the amount ratio of D-fructose produced and D-glucose added.

For potential industrial applications, the temperature effect on the conversion efficiency of CbGI was determined by measuring the fructose yields as described above after incubations at 70°C and 80°C for various durations with 2 M D-glucose as the substrate. The conversion rate of CbGI was estimated by increasing the concentrations of D-glucose from 0.1 M to 3 M at 80°C. The amounts of D-fructose produced were determined the HPLC chromatographic method as described above. The measurements were carried out in three independent experiments, and each experiment included triplicate.

## 3. Results and Discussion

### 3.1. Gene Cloning, Expression, and Purification

The ORF of CbGI contained 1317 bp, which coded for a polypeptide of 438 amino acid residues. The comparison of the CbGI amino acid sequence with other sequence proteins using Blast P showed a similarity of about >95% with many putative xylose isomerases from the genus of *Caldicellulosiruptor* (accession numbers: WP_013430944.1, WP_013403911.1, WP_013433194.1, and WP_013411463.1). It exhibited the highest identity of 84% with the GI derived from *Thermoanaerobacter ethanolicus* CCSD1 (accession number: AKM94132.1), which has been characterized [4], and showed 77% identity with *Thermoanaerobacterium thermosulfurigenes* (1A0C_A) in the PDB database.

**Figure 1:**
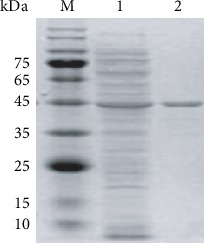
SDS-PAGE analysis of the purified recombinant CbGI. M: the molecular mass standards; 1: the crude enzyme; 2: the purified recombinant CbGI.

For expression in *E. coli*, the CbGI was successfully ligated into the pET-30a (+) vector by overlapping PCR. After IPTG induction and cell disruption, significant GI activity was detected in the crude enzyme. Following a single step of Ni^2+^ chelate affinity chromatography, the recombinant CbGI was purified to be on electrophoretic homogeneity presented in Figure 1. The apparent protein molecular weight was estimated to be 45.0 kDa, which is consistent with the theoretical value.

### 3.2. Enzymatic Properties of Purified Recombinant CbGI

**Figure 2:**
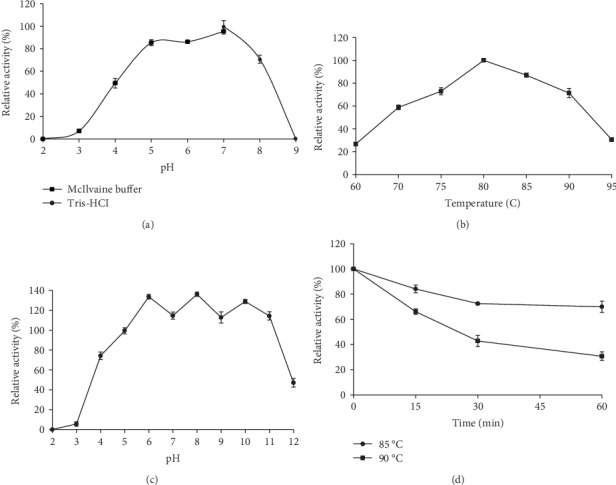
The enzymatic properties of purified recombinant CbGI. (a) Effect of pH on enzyme activity determined at 80°C for 30 min. (b) Effect of temperature on enzyme activity determined at pH 7.0 for 30 min. (c) pH stability. The residual activities were determined under standard conditions (80°C and pH 7.0 for 30 min) after 1 h incubation at 37°C and different pHs. (d) Thermostability. The residual activities were determined under standard conditions (80°C and pH 7.0 for 30 min) after incubation at pH 7.0 and 85°C or 90°C for various durations.

The optimum pH for CbGI activity was determined to be pH 7.0 presented in Figure 2(a), such as GI from *T. saccharolyticum* [9], *Actinoplanes missouriensis CICIM B0118(A)* [10], and *Streptomyces griseus* [11]. The optimum pH of these strains is between 7.0 and 7.5. For the isomerization reaction of glucose to fructose, the pH has an important effect on the conversion rate of fructose. Industrial applications require a slightly acidic pH environment to reduce by-products and browning reactions under alkaline conditions [12]. However, most commercial GIs have an optimum pH between 7.5 and 9.0, and the enzyme activity is greatly reduced under acidic conditions. Although the optimum pH of CbGI is 7.0, the enzyme displayed adaptability over a broad pH range, showing more than 50% of the maximum activity at pH 4.0 to 8.0 and 88% activity at pH 5.0. This may be solved by using acidic reaction. CbGI had an optimum temperature of 80°C presented in Figure 2(b) and retained more than 50% of maximum activity at 70°C–90°C. The optimum temperature for most GIs is between 60°C and 90°C. For example, the optimum temperature of a class II GI from the thermophilic *Caldicoprobacter algeriensis* was 90°C [13], GI from *Thermobifida fusca WSH03-11* is expressed in *E. coli* with an optimum temperature of 80°C [6]. The optimum temperature of CbGI was higher than that from *T. thermosulfurigenes* (65°C) [14]. In recent years, due to the urgent demand for high-temperature GI, many ultra-high-temperature enzymes have been cloned and expressed, such as GI from *T. ethanolicus CCSD1 (TEGI)*, which had an optimum temperature of 90°C [4], and *T. neapolitana 5068* GI with optimum temperature of between 95 and 100°C [15]. Also, by gene mining, the optimum temperature for GI from *Thermus oshimai* is as high as 95°C [7]. It has been reported that, theoretically, 55% conversion can be achieved when the isomerization temperature is above 85°C [13, 16]. Although the optimum temperature for many GIs is above 90°C, excessive temperatures for production consume energy and increase production costs [6]. In addition, ultra-high temperature also easily deteriorates the substrates and produces more by-products [12]. The study on the pH stability of CbGI showed that the enzyme was quite stable over a broad pH range presented in Figure 2(c). After incubation at 37°C for 1 h, the enzyme retained 74%–114% of the initial activity at pH 4.0–11.0. Moreover, CbGI displayed good stability at 85°C and below, retaining more than 72% of its initial activity after 1 h incubation presented in Figure 2(d). This thermophilic GI with neutral pH stability and thermostability may have application potentials in high-temperature industries.

### 3.3. Kinetics of CbGI

The kinetic parameters were calculated using the Lineweaver-Burk plots. The *K*m, *V*max, and *k*cat/*K*m values of CbGI were determined to be 42.61 mM, 8.22 U/mg, and 9.69/s/mM, respectively. In comparison to other reported counterparts, CbGI had a lower *K*m and a higher *k*cat/*K*m value. It indicated that CbGI has superior substrate affinity and higher catalytic efficiency.

### 3.4. Effect of Metal Ions and Chemical Reagents on CbGI

It has been reported that divalent cations like Co^2+^, Mg^2+^, or Mn^2+^ can activate the enzymatic activity of GIs [17]. For CbGI, an increase in relative activity until 158% was noted when Co^2+^ was added at the final concentration of 0.5 mM. Although Mg^2+^ and Co^2+^ are essential for GI activity, they play different roles: Mg^2+^ is superior to Co^2+^ as an activator, while Co^2+^ plays a role in stabilizing the enzyme [12]. Such GI derived from *S. olivaceoviridis E-86* significantly increased enzyme activity when Mg^2+^ was added [18]. The glucose isomerase derived from the *T. strain B6A* requires Mg^2+^ and Co^2+^ [19], and *S*. sp. SK GI mutant G219D is converted from Co^2+^ dependent to Mn^2+^ dependent [20]. Therefore, the effects of metal ions and chemical reagents on CbGI activity were determined in the presence of 10 mM Co^2+^ presented in Table 2. K^+^ and *β*-mercaptoethanol had no effect on the enzyme activity (less than 10%). Other metal ions and chemicals including Cr^3+^, Ni^2+^, Mn^2+^, Ca^2+^, SDS, and EDTA strongly inhibited the CbGI activity by 50% and more.

### 3.5. Conversion Efficiency of CbGI

To assess the conversion efficiency of CbGI, 30 U, 60 U, or 90 U of CbGI was added into the 3 mL reaction systems containing 1 M of D-glucose and 5 mM Co^2+^. Since no difference was detected in the conversion rates (data not shown), 60 U of CbGI was used for the following conversion efficiency assays.

**Figure 3:**
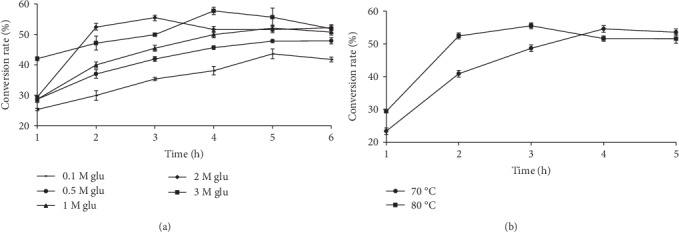
Conversion efficiency of CbGI with D-glucose as the substrate. (a) Conversion rates of CbGI against different concentrations of D-glucose. (b) Conversion rate of CbGI at different temperatures.

To investigate the conversion efficiency of CbGI at different temperatures, the reactions were conducted at pH 7.0 and 70°C or 80°C, respectively, with 2 M of glucose as the substrate. The results showed that the conversion rates of CbGI at 70°C and 80°C were similar presented in Figure 3(b), but the time reaching equilibrium was different. The reactions at 80°C reached equilibrium within 3 h, while 4 h at 70°C. The other thermophilic GIs were reported to obtain high bioconversion rate at high temperature. Such as the maximal bioconversion rate of 54.7% was accomplished by GICA from *C. algeriensis* at 85°C for 3 h [13], which 52.2% was achieved by TnapXI from *T. naphthophila* RKU-10 [21].

The time course of D-glucose conversion into D-fructose by CbGI was determined at 80°C and pH 7.0 with 0.1-3 M D-glucose as the substrate. The results showed that the conversion rate of CbGI increased along with the increased concentration of D-glucose. The maximum conversion rate, 57.3%, was observed at 4 h when 3 M D-glucose was used as the substrate (*w*/*v* = 54%) presented in Figure 3(a). This is the first time that a conversion of more than 55% is achieved under high concentration substrates compared to previous reports. It is reported that the GI from *T. oshimai* strain had a conversion of 52.16% < 55% at a substrate concentration of 400 g/L [7], and this study may have analyzed that the isomerization temperature is too high and leads to the formation of more by-products. This affects the yield of fructose, a small part of glucose will be degraded during the isomerization reaction, producing some by-products such as mannose, psicose, and acidic compounds. The GI derived from *Thermobifida fusca WSH03-11* has a maximum conversion of 53% at a 45% (mass-to-volume) glucose concentration [6]. The GI from *Streptomyces* sp. has a conversion rate of 55% at a low glucose concentration (mass-to-volume ratio of 30%) [18]. The wild-type and mutant GIs from *T. ethanolicus* have a conversion rate of 53.8% and 55.4% at 10% glucose concentration, respectively [4]. However, industrial production requires a slightly acidic GI, which not only reduces the formation of by-products but also does not require adjustment of the pH of the reaction system from the scarification process to the isomerization process, reducing energy consumption [1]. In these reports, most GIs did not achieve a conversion of 55%, some enzymes achieved a conversion of 55% at low-glucose concentrations, and there was no description of the relationship between conversion and substrate concentration. The results of this study showed that the conversion rate of CbGI increased with the increase of substrate concentration, which is somewhat different from previous reports, such as the conversion rate of GI from *Thermus oshimai* increased first and then decreased [22]. This indicates that CbGI has strong substrate tolerance. CbGI achieves a high conversion rate of 52.7% at a glucose concentration of 3 M, which can effectively reduce costs both in the production of high-fructose syrup and in the formation of its product (70% dry matter).

## 4. Conclusions

In this study, the gene cloning and expression of a novel GI from *C. bescii* was characterized. The CbGI was neutral and thermophilic, with excellent thermal and pH stability. Moreover, the enzyme had high-glucose conversion rate of 57.3% with 3 M glucose as the substrate. CbGI is favorable for industrial one-step production of HFCS-55 at a cost-effective way.

## Figures and Tables

**Table 1:** Primers used for PCR amplification.

Primer name	Sequence (5′⟶3′)
CbGI-pET30a-F	TTTAAGAAGGAGATATACATATGAAGTACTTCAAAGACATTCCAGAAGTAAAATA
CbGI-pET30a-R	CAGTGGTGGTGGTGGTGGTGTTATTCGCTGAACATATATTTGTTCAAAATCATCT
pET30a-CbGI-F	AATATATGTTCAGCGAATAACACCACCACCACCACCACTGAGATCCG
pET30a-CbGI-R	ATGTCTTTGAAGTACTTCATATGTATATCTCCTTCTTAAAGTTAAACAAAATTAT

**Table 2:** Effects of different metal ions and chemical reagents on the enzyme activity of CbGI.

Chemicals	Relative activity (%)	Chemicals	Relative activity (%)
None (Co^2+^)	100 ± 2.9	None (Co^2+^)	100 ± 0.9
K^+^	111 ± 1.5	Mg^2+^	117 ± 2.9
Na^+^	101 ± 2.8	*β*-Mercaptoethanol	107 ± 2.1
Mg^2+^	98 ± 1.4	K^+^	94 ± 2.4
*β*-Mercaptoethanol	79 ± 1.2	Na^+^	86 ± 3.3
Mn^2+^	29 ± 3.2	Zn^2+^	73 ± 2.6
Cr^3+^	12 ± 2.6	Fe^2+^	57 ± 3.2
Ca^2+^	12 ± 0.5	Ca^2+^	49 ± 2.6
Ni^2+^	9 ± 1.4	SDS	43 ± 1.0
SDS	7 ± 1.0	Mn^2+^	32 ± 1.1
Ag^+^	1 ± 0.5	Ni^2+^	11 ± 3.2
EDTA	ND	Cr^3+^	3 ± 0.2
Fe^2+^	ND	EDTA	ND

*Note.* ND: no enzyme activity detected.

## Data Availability

The data used to support the findings of this study are available from the corresponding author upon request.
